# Cysteine-based protein folding modulators for trapping intermediates and misfolded forms†

**DOI:** 10.1039/d2ra04044a

**Published:** 2022-09-21

**Authors:** Hayato Nishino, Mai Kitamura, Shunsuke Okada, Ryosuke Miyake, Masaki Okumura, Takahiro Muraoka

**Affiliations:** Department of Applied Chemistry, Graduate School of Engineering, Tokyo University of Agriculture and Technology 2-24-16 Naka-cho, Koganei Tokyo 184-8588 Japan muraoka@go.tuat.ac.jp; Department of Chemistry and Biochemistry, Graduate School of Humanities and Sciences, Ochanomizu University 2-1-1 Otsuka, Bunkyo-ku Tokyo 112-8610 Japan; Frontier Research Institute for Interdisciplinary Sciences, Tohoku University 6-3 Aramaki-Aza-Aoba, Aoba-ku Sendai 980-8578 Japan; Institute of Global Innovation Research, Tokyo University of Agriculture and Technology 3-8-1 Harumi-cho, Fuchu Tokyo 183-8538 Japan; Kanagawa Institute of Industrial Science and Technology (KISTEC) Kanagawa 243-0435 Japan

## Abstract

Folding is a key process to form functional conformations of proteins. Folding *via* on-pathway intermediates leads to the formation of native structures, while folding through off-pathways affords non-native and disease-causing forms. Trapping folding intermediates and misfolded forms is important for investigating folding mechanisms and disease-related biological properties of the misfolded proteins. We developed cysteine-containing dipeptides conjugated with amino acids possessing mono- and diamino-groups. In oxidative protein folding involving disulfide-bond formation, the addition of cysteine and oxidized glutathione readily promoted the folding to afford native forms. In contrast, despite the acceleration of disulfide-bond formation, non-native isomers formed in significantly increased yields upon the addition of the dipeptides. This study provides a molecular design of cysteine-based protein-folding modulators that afford proteins adopting non-native conformations through intermolecular disulfide-bond formation. Because of the intrinsic reversibility of the disulfide bonds upon redox reactions, the disulfide bond-based approach demonstrated here is expected to lead to the development of reversible methodologies for trapping transient and misfolded forms by intermolecular disulfide bond formation and restarting the folding processes of the trapped forms by subsequent cleavage of the intermolecular disulfide bonds.

## Abstract 2

In this study, cysteine-containing dipeptides conjugated with amino acids possessing mono- and diamino-groups were developed as protein-folding modulators affording non-native forms through intermolecular disulfide-bond formation.

## Graphical abstract

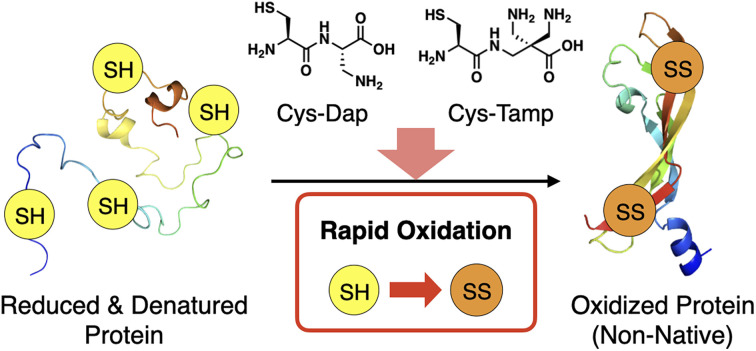

## Introduction

The folding process of a polypeptide chain influences the fate of the protein.^1,2^ Folding *via* stepwise conversion among on-pathway intermediates allows for the formation of the native conformation of the protein with its biological activity.^3–9^ Meanwhile, an off-pathway folding process forming non-native intermediates affords misfolded and even pathological isomers of the protein.^10–12^ For instance, side effects of pharmacological administration for psychiatric disorders induce aggregation-prone proinsulin containing non-native disulfide bonds, leading to type-II diabetes.^13^ To investigate the biological as well as disease-related properties of a protein, it is necessary to develop methodologies of not only folding promotion to the native form but also folding-intermediates trapping.^14^ In the oxidative protein folding reactions involving disulfide-bond formation, reactions of cysteine residues are one of the major factors that regulate the folding process.^15,16^ Namely, disulfide-bond formation between the native cysteine–residue pairs allows for folding of the polypeptide chain to the native conformation, while folding through disulfide bonding between non-native cysteine–residue pairs results in the formation of misfolded isomers.

**Fig. 1:**
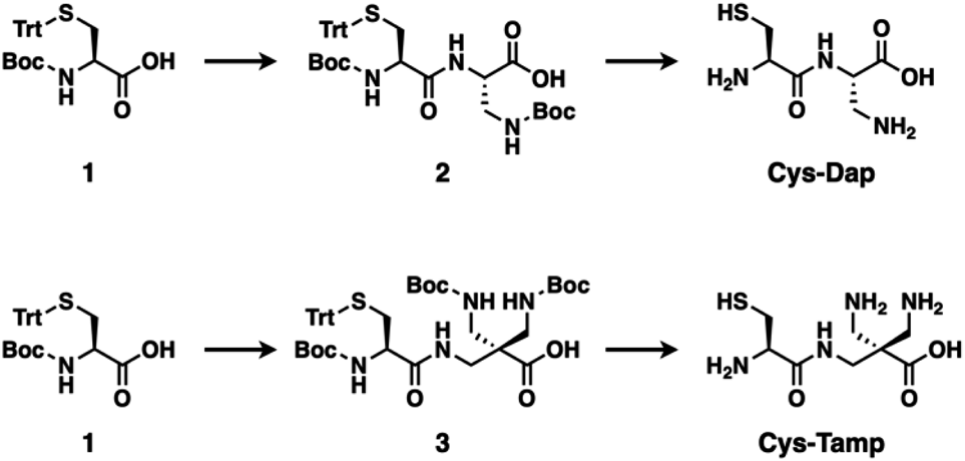
Synthetic schemes of Cys-Dap and Cys-Tamp.

For the regulation of protein folding, chemical approaches are effective.^17,18^ In the endoplasmic reticulum, enzymes such as protein disulfide isomerase promote disulfide-bond shuffling by cleavage and re-formation of disulfide bonds to encourage the structural conversion of substrate proteins into thermodynamically stable conformations, *i.e.*, the native structure.^19^ Inspired by the enzymatic process, a variety of compounds such as thiols with increased acidity, glutathione derivatives, and selenols have been developed for promotion of protein folding.^20–25^ On the other hand, methodologies that can trap folding intermediates and misfolded forms are limited to genetic mutations^26–28^ and chemical treatments using iodoacetic acid and maleimide-appending compounds.^29,30^ Here, we demonstrate that newly developed cysteine-based dipeptides, Cys-Dap and Cys-Tamp (Fig. 1), facilitated the oxidation of proteins and afforded isomers with non-native conformations preferentially through intermolecular disulfide-bond formation.

## Results and discussion

### Synthesis of cysteine-containing dipeptides

The cysteine-containing dipeptides, Cys-Dap and Cys-Tamp, were synthesized from cysteine protected with Boc and trityl groups in a straightforward approach (Fig. 1). Boc- and trityl-protected cysteine 1 was coupled with Boc-protected (*S*)-diaminopropionic acid (Dap) to afford 2, and subsequent deprotection of Boc and trityl groups provided Cys-Dap. Cys-Tamp was synthesized from compound 3 that was produced by coupling 1 and 3-amino-2,2-bis{[(*tert*-butoxycarbonyl)amino]methyl} propanoic acid (Boc-protected Tamp).^31,32^

### Effects of cysteine-containing dipeptides on disulfide-bond formation of RNase A

**Fig. 2:**
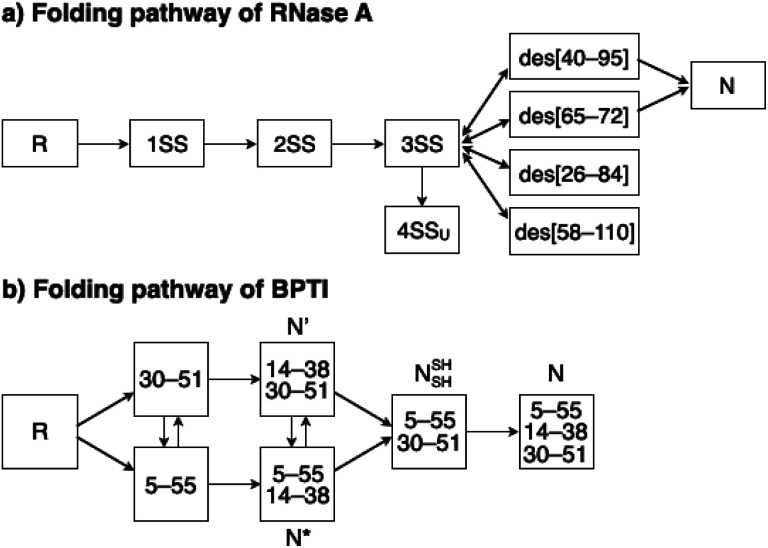
Folding pathways of (a) RNase A and (b) BPTI. R and N denote reduced and native forms, respectively. In (a), 1SS, 2SS, 3SS: folding intermediates containing one, two, and three disulfide bonds, respectively; 4SS_U_: non-native forms with four disulfide bonds; des[M–N]: a folding intermediate with three native disulfide-bond pairings lacking the Cys_M_–Cys_N_ disulfide bond. In (b) N′, N*, N^SH^_SH_: folding intermediates with two disulfide bonds at Cys14–Cys38 and Cys30–Cys51, Cys5–Cys55 and Cys14–Cys38, and Cys5–Cys55 and Cys30–Cys51, respectively.

To investigate the effects of the cysteine-containing dipeptides on the regulation of oxidative protein folding, folding reactions of ribonuclease (RNase) A were analyzed. RNase A forms four disulfide bonds in its native form (N), and the reduced form (R) folds into N by consecutive disulfide-bond formation through 1SS, 2SS, and 3SS intermediates containing one, two, and three disulfide bonds, respectively (Fig. 2a).^5,33–35^ As a side reaction, 4SS_U_, non-native forms with four disulfide bonds, can be produced from the 3SS intermediate.

**Fig. 3:**
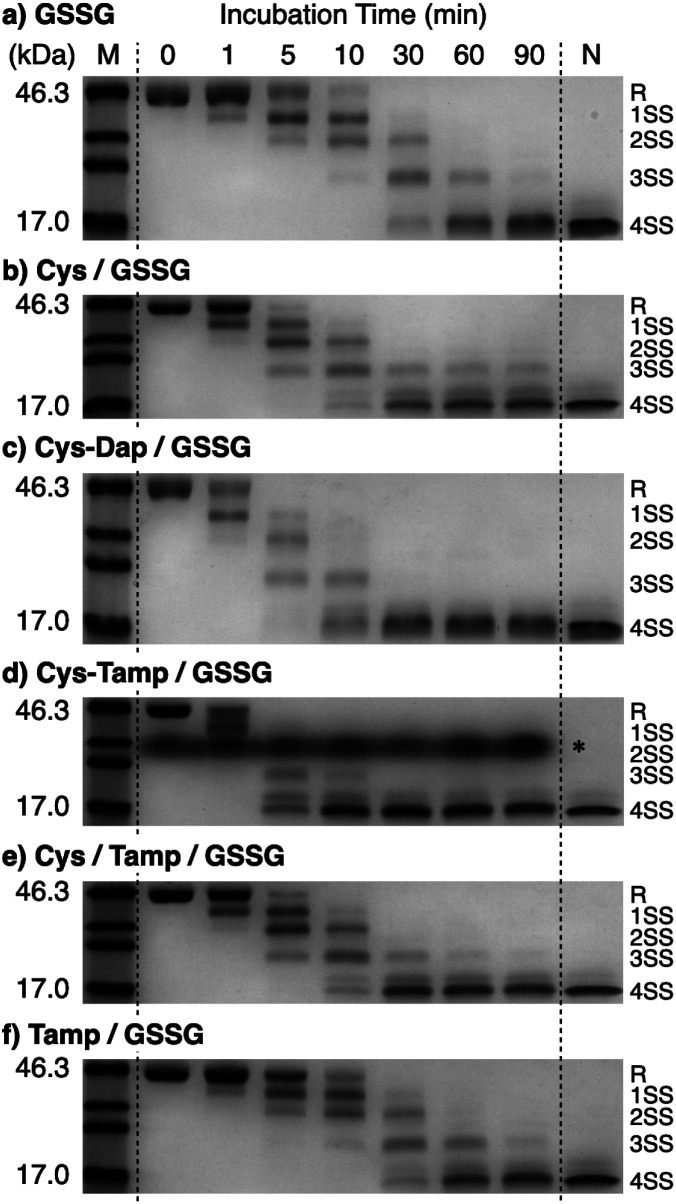
Time course analyses of RNase A oxidation by SDS-PAGE. SDS-PAGE gel images monitoring the oxidation of RNase A (8.0 μM) in the presence of (a) GSSG, (b) cysteine and GSSG, (c) Cys-Dap and GSSG, (d) Cys-Tamp and GSSG, (e) cysteine, Tamp and GSSG, and (f) Tamp and GSSG (thiol compounds and Tamp: 1.0 mM; disulfide compounds: 0.20 mM) in a buffer (50 mM Tris–HCl, 300 mM NaCl, pH 7.5). The oxidation reactions were quenched with malPEG-2000 after 1, 5, 10, 30, 60, and 90 min incubations. The leftmost and rightmost lanes show the bands corresponding to the markers (M) and native RNase A (N), respectively. The bands marked with an asterisk in (d) were also observed under the same condition regardless of the absence of RNase A (see also Fig. S1 in ESI†). The bands marked with an asterisk in (d) are assigned to a complex of SDS and Cys-Tamp (see also Fig. S1 in ESI†). R represents reduced form, and 1SS, 2SS, 3SS, and 4SS represent species containing one, two, three, and four disulfide bonds, respectively. In these species, conjugates between RNase A and thiol-additives can be included.

**Fig. 4:**
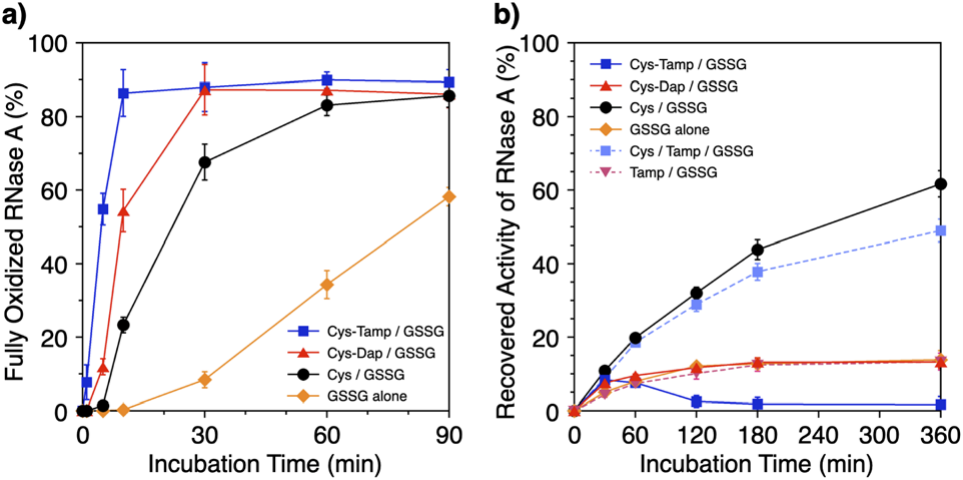
(a) Time-course changes of the percentages of fully oxidized RNase A (8.0 μM) and (b) recovered enzymatic activity of RNase A (8.0 μM) in the presence of (blue squares) Cys-Tamp and GSSG, (red triangles) Cys-Dap and GSSG, (black circles) cysteine and GSSG, (orange diamonds) GSSG alone, (light blue squares) cysteine, Tamp and GSSG, and (pink inverted triangles) Tamp and GSSG (thiol compounds and Tamp: 1.0 mM; disulfide compounds: 0.20 mM) in a buffer (50 mM Tris–HCl, 300 mM NaCl, pH 7.5). The percentages of 4SS were quantified by SDS-PAGE analyses (original data: Fig. 3). The activity was evaluated by spectroscopic monitoring of the hydrolysis of cCMP to 3′-CMP at 30 °C. Error bars indicate the means ± SEM of three independent experiments.

Disulfide-bond formation of reduced RNase A was conducted by oxidized glutathione (GSSG) as an oxidant, and the reaction was monitored by electrophoresis. Treatment of the reaction mixture with malPEG-2000 (average *M*_n_ = 2000), which reacts with free thiol groups on RNase A, allows for the separation of the folding intermediates by sodium dodecyl sulfate-polyacrylamide gel electrophoresis (SDS-PAGE). In the presence of GSSG, reduced RNase A transformed into 1SS, 2SS, and 3SS intermediates over the incubation time, and a band corresponding to 4SS (N + 4SS_U_) appeared after 30 min incubation (Fig. 3a). In the presence of the cysteine and GSSG mixture, a band corresponding to 4SS was observed after 10 min incubation (Fig. 3b). Interestingly, the disulfide-bond formation proceeded even faster in the presence of the cysteine-containing dipeptides and GSSG than in the other conditions. Particularly, in the presence of Cys-Tamp and GSSG, the band of 4SS appeared after 5 min incubation (Fig. 3d). A quantitative analysis of the fully oxidized RNase A formation based on the SDS-PAGE analyses shows that nearly 90% conversion from R was completed after 10 min incubation in the condition of Cys-Tamp and GSSG (Fig. 4a, blue squares). The condition of cysteine and GSSG required 90 min incubation for 90% conversion, while the oxidation proceeded more slowly in the presence of GSSG alone (Fig. 4a, black circles and orange diamonds). Importantly, the mixtures of cysteine, Tamp, and GSSG or Tamp and GSSG did not accelerate the oxidation compared to the Cys-Tamp and GSSG mixture (Fig. 3e and f). Therefore, the covalent conjugation of Tamp largely influenced the properties of the cysteine thiol group, enabling rapid disulfide-bond formation.

### Effects of cysteine-containing dipeptides on folding of RNase A to native and misfolded forms

The efficiency of oxidative refolding of RNase A to its native form was investigated by the recovery of enzymatic activity (Fig. 4b). In the presence of GSSG, RNase A showed an increase in the enzymatic activity during the initial 120 min incubation to reach a plateau of 12% recovery. The cysteine/GSSG system showed an enhanced recovery of the RNase A activity to 62% after 360 min incubation. Interestingly, the Cys-Dap/GSSG system resulted in a significant drop in the recovery yield to 12%, and the Cys-Tamp/GSSG system hardly showed any recovery of RNase A activity (<2%). These results indicate that coupling mono- and diamino-groups lowers the efficiency of cysteine to promote the protein refolding to the native form. As indicated in Fig. 4a, the Cys-Dap/GSSG and Cys-Tamp/GSSG systems facilitated the disulfide-bond formation of reduced RNase A more quickly than the cysteine/GSSG system. Therefore, it is likely that cysteine coupled with mono- and diamino-groups produced non-native isomers of RNase A in the oxidation reactions driven by GSSG.

To investigate the effects of Tamp on the refolding reaction, enzymatic activity recoveries of RNase A in the Cys/Tamp/GSSG and Tamp/GSSG systems were measured. In the Tamp/GSSG system, reduced RNase A showed an analogous activity recovery profile compared to that in GSSG alone. Meanwhile, in the Cys/Tamp/GSSG system, reduced RNase A readily recovered its enzymatic activity up to 49% after 360 min incubation. These results suggest that simply adding Tamp to the reaction condition has limited influence on the refolding process of RNase A.

### Effects of cysteine-containing dipeptides on BPTI folding

**Fig. 5:**
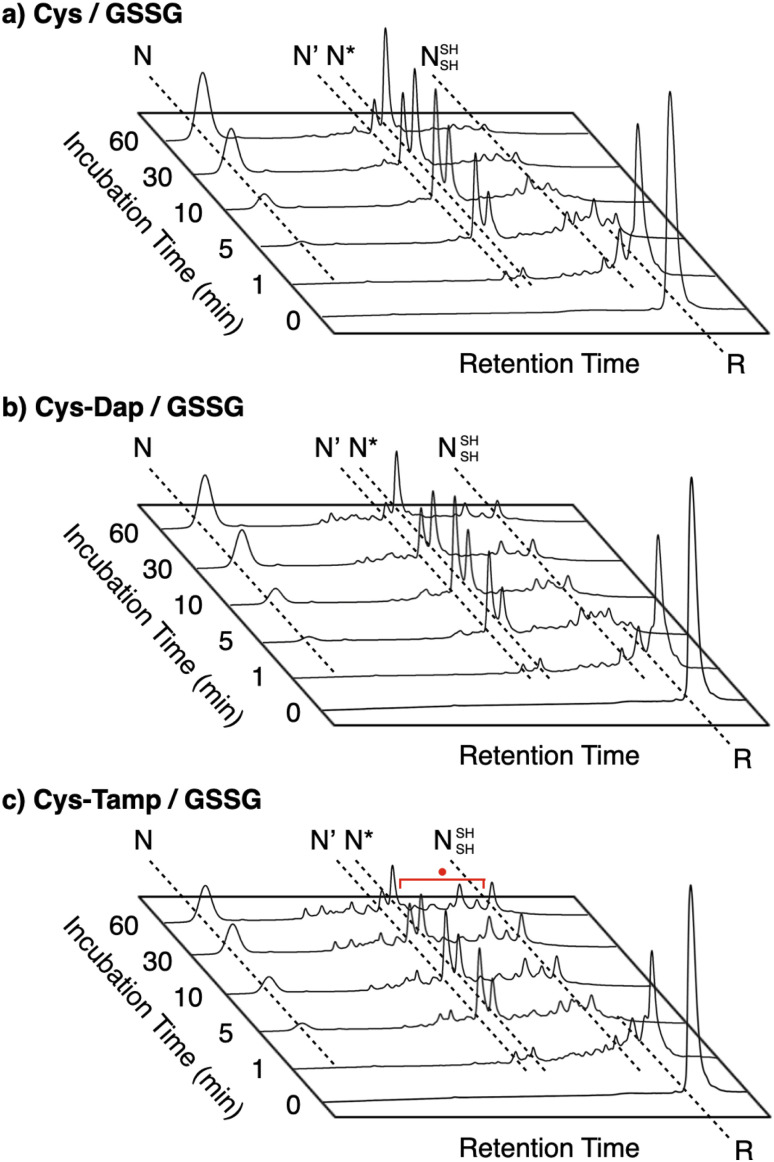
Time-course reversed-phase HPLC traces of oxidative folding of BPTI (30 μM) in the presence of GSSG (0.20 mM) and (a) cysteine, (b) Cys-Dap, and (c) Cys-Tamp (1.0 mM) between 16 and 45 min retention time. N and R depict native and reduced forms of BPTI, respectively. The structural information of N′, N*, and N^SH^_SH_ is indicated in the caption of Fig. 2. Eluents: water (containing 0.05% TFA) and CH_3_CN (containing 0.05% TFA) with a linear gradient (water/CH_3_CN = 80/20 at 16 min to 65/35 at 45 min); flow rate: 1.0 mL min^−1^; detection wavelength: 229 nm.

To gain a more general view of the effects of the cysteine-containing dipeptides on the protein folding reaction, we studied the folding reactions of bovine pancreatic trypsin inhibitor (BPTI). BPTI possesses three disulfide bonds, *i.e.*, C_5_–C_55_, C_14_–C_38_ and C_30_–C_51_, and forms quasi-native intermediates such as N′ and N* with two disulfide bonds in the on-pathway folding process to afford the native structure (N, Fig. 2b).^36^ Oxidative folding of reduced and denatured BPTI (R, 30 μM) was monitored by reversed-phase high-performance liquid chromatography (HPLC). As we reported previously, R-BPTI showed spontaneous folding into N in the presence of GSSG as an oxidant.^24^ The HPLC analysis indicated that the fraction of R disappeared after 30 min incubation and N-BPTI formed in 21% yield after 60 min. In the cysteine/GSSG system, the folding reaction of BPTI to its N form proceeded faster, where R disappeared within the initial 10 min incubation and the yield of N after 60 min was enhanced to 38% (Fig. 5a). By using Cys-Dap or Cys-Tamp as a thiol additive, the oxidation reaction of R-BPTI was further accelerated. Namely, in the Cys-Dap/GSSG and Cys-Tamp/GSSG systems, the fractions of R disappeared within 5 min. Interestingly, despite such rapid oxidation reactions, the yields of N were lower than that observed in the cysteine/GSSG system (Cys-Dap/GSSG: 30%; Cys-Tamp/GSSG: 18%). Furthermore, the HPLC profile changed slowly, particularly after 5 min incubation in the Cys-Tamp/GSSG system, and the HPLC trace after 60 min incubation was rather complicated with some fractions that usually did not appear (fractions marked with a red circle and bracket in Fig. 5c). These results indicate that R-BPTI transformed into multiple isomers by rapid disulfide-bond introduction and less efficient disulfide-bond shuffling in the Cys-Tamp/GSSG condition. The complicated HPLC profile indicates the formation of structural isomers of BPTI including not only on-pathway folding intermediates but also off-pathway species.^37,38^ It should also be noted that, analogous to the case of RNase A folding, addition of Tamp hardly influenced the folding process of BPTI. The yields of N-BPTI after 60 min incubation were 23% in the Tamp/GSSG system and 41% in the Cys/Tamp/GSSG system (Fig. S2†).

### Discussion of chemical properties of cysteine-containing dipeptides on the folding process

**Fig. 6:**
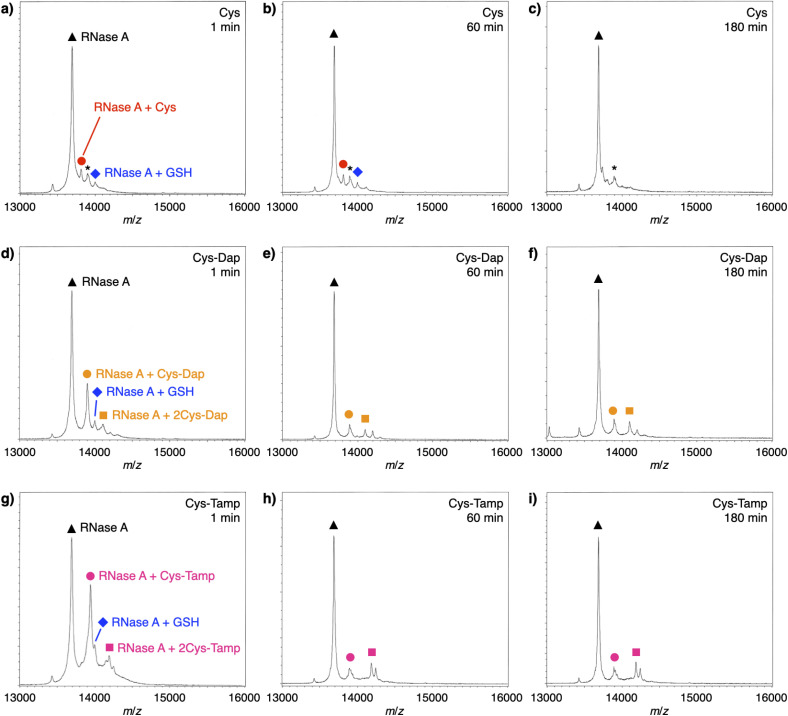
Time-course MALDI-TOF MS analyses of RNase A (8.0 μM) in the presence of (a–c) cysteine and GSSG, (d–f) Cys-Dap and GSSG, and (g–i) Cys-Tamp and GSSG at 1, 60 and 180 min of incubation after the addition of the thiol and disulfide compounds (thiol compounds: 1.0 mM; disulfide compounds: 0.20 mM) in a buffer (50 mM Tris–HCl, 300 mM NaCl, pH 7.5). Matrix: sinapinic acid. Linear and positive modes. Signals marked with black triangles, blue diamonds, red circles, orange circles, orange squares, pink circles, pink squares, and asterisks correspond to RNase A, RNase A–GSH conjugate, RNase A–cysteine conjugate, RNase A–Cys-Dap conjugate, RNase A–(Cys-Dap)_2_ conjugate, RNase A–Cys-Tamp conjugate, RNase A–(Cys-Tamp)_2_ conjugate, and RNase A–sinapinic acid complex, respectively.

To investigate the reactivities of the thiol groups in the oxidative protein folding, chemical properties, *i.e.*, acidity (p*K*_a_) and redox potential (*E*^0^’), of the cysteine-based dipeptides were evaluated. The properties of cysteine thiol group were reported as p*K*_a_ = 8.37 and *E*^0^’ = −220 mV.^39^ The p*K*_a_ values were lowered by increasing the number of amino groups in the molecules: p*K*_a_ = 6.83 ± 0.09 (Cys-Dap) and 6.67 ± 0.09 (Cys-Tamp).§§p*K*_a_ values of cysteine in the presence of Dap and Tamp were 8.36 ± 0.03 and 8.27 ± 0.04, respectively, indicating that the acidity of cysteine is hardly influenced by Dap or Tamp. Measurement condition: [cysteine] = [Dap] = [Tamp] = 125 μM. The *E*^0^’ values also decreased to −256 ± 8 mV (Cys-Dap) and −281 ± 1 mV (Cys-Tamp). The decreased *E*^0^’ values indicate that Cys-Dap and Cys-Tamp prefer the oxidized states to cysteine, so that, in the oxidative reaction by GSSG, intermolecular disulfide bond formation between a protein and a dipeptide would be preferential to the intramolecular disulfide bond formation between cysteine residues in a protein. This electrochemical property is likely the major reason of the faster disulfide bond formation of proteins observed in the presence of dipeptides. The increased acidity of Cys-Dap and Cys-Tamp relative to cysteine suggests enhanced nucleophilicity of the thiol units. Nucleophilic reactions of Cys-Dap and Cys-Tamp to cleave an intramolecular disulfide bond between cysteine residues in a protein can also provide protein–dipeptide conjugates with intermolecular disulfide bonds. Indeed, in the RNase A folding reactions, the formation of folding intermediates conjugated with the dipeptides was suggested by matrix-assisted laser desorption/ionization time-of-flight mass (MALDI-TOF MS) spectrometry. In the presence of cysteine and GSSG, a signal assigned to a one-to-one conjugate between RNase A and cysteine was observed after 1 min incubation (RNase A + Cys, red circle in Fig. 6a). After 180 min incubation, the signal of the conjugate could not be identified clearly due to significantly weakened intensity (Fig. 6c). In contrast, in the presence of Cys-Dap or Cys-Tamp, signals assigned to one-to-one and even one-to-two conjugates between RNase A and the dipeptides were clearly observed after 180 min incubation (orange circle and square in Fig. 6f and pink circle and square in Fig. 6i). Although quantitative comparisons are technically difficult based on the mass spectroscopic signal intensities, this result suggests relatively long lifetimes of the conjugates between RNase A and the dipeptides forming intermolecular disulfide bonds. Thus, the folding intermediates and non-native forms could be stabilized.

## Experimental

### Concentration determination of thiol compounds

A thiol compound dissolved in 10 mM HCl aq. was diluted in a buffer (50 mM Tris–HCl, 0.3 M NaCl, pH 7.5). The mixture was added to an aqueous solution of 5,5′-dithiobis(2-nitrobenzoic acid) (DTNB). The concentration of the thiol compound was determined by the absorbance at 412 nm measured at 30 °C.^40^

### Preparation of reduced and denatured RNase A

RNase A (from bovine pancreas) dissolved in a buffer (200 mM Tris–HCl, pH 8.6) containing 6.0 M guanidium chloride and 100 mM dithiothreitol (DTT) was incubated for 2 h at 25 °C. The mixture was dialyzed for 2 h at 25 °C with 10 mM HCl aq., and the dialysis was performed three times. The content of the secondary structure after the dialysis was characterized by the circular dichroism spectroscopic measurement (Fig. S3†).

### SDS-PAGE measurements of RNase A

A buffer (50 mM Tris–HCl, 0.3 M NaCl, pH 7.5) containing oxidized glutathione (GSSG, 0.20 mM) and a thiol compound (1.0 mM) was preincubated for 10 min at 30 °C.^41^ Reduced and denatured RNase A (final concentration: 8.0 μM) was added to the buffer, and the mixture was incubated for 90 min at 30 °C. During the incubation, 16 μL of the reaction mixture was collected at designated times (0, 1, 5, 10, 30, 60, and 90 min). The collected mixture was added to Laemmli's ×4 SDS-loading buffer^42^ (16 μL) containing malPEG-2000 (10 mM) to quench disulfide bond formation of free thiol groups. Redox states of RNase A obtained by the above treatment were separated by non-reducing 14% SDS-PAGE using WIDE RANGE gel (Nacalai Tesque, Kyoto, Japan). Proteins were visualized by staining with Coomassie brilliant blue G-250. The band intensities were analyzed by a ChemiDoc Touch imaging system and Image Lab (Bio-Rad, Hercules, CA, USA).

### Enzymatic activity measurements of RNase A

A buffer (50 mM Tris–HCl, 0.3 M NaCl, pH 7.5) containing oxidized glutathione (GSSG, 0.20 mM) and a thiol compound (1.0 mM) was preincubated for 10 min at 30 °C. Reduced and denatured RNase A (final concentration: 8.0 μM) was added to the buffer, and the mixture was incubated for 360 min at 30 °C. During the incubation, 110 μL of the reaction mixture was collected at designated times (30, 60, 120, 180, and 360 min). The collected mixture was added to a buffer (50 mM Tris–HCl, 0.3 M NaCl, pH 7.5, 330 μL) containing cCMP (final concentration: 0.80 mM). The mixture was poured into a quartz cuvette to monitor the change in absorption intensity at 284 nm at 30 °C for 4 min by a V-650 UV-vis spectrophotometer (JASCO, Tokyo, Japan). Values represent means ± SEM from three independent experiments.

### BPTI folding assay

Reduction and denaturation of BPTI (from bovine lung) was carried out as follows. BPTI (10 mg) was dissolved in 1 mL of 0.1 M Tris–HCl (pH 8.0) containing 30 mM DTT and 8 M urea and the solution was allowed to stand for 3 h at 50 °C. The reduced and denatured protein was purified by HPLC using an InertSustain C18 column (*φ*4.6 × 250 mm, GL Sciences, Tokyo Japan), lyophilized and stored at −30 °C until use. Purified reduced/denatured BPTI was analyzed by MALDI-TOF MS, resulting in the confirmation of disulfide bonds reduction. For the assay, fully reduced and denatured BPTI (600 μM) was dissolved in 6 M urea containing 0.05% trifluoroacetic acid. A mixture of GSSG and a thiol compound was dissolved in a buffer (50 mM Tris–HCl, 300 mM NaCl, pH 7.5), and the resulting mixture was preincubated for 10 min at 30 °C. To the preincubated mixture, the solution of reduced and denatured BPTI was added (final concentrations: BPTI: 30 μM, urea: 300 mM, GSSG: 200 μM, thiol compound: 1.0 mM, Tris–HCl: 50 mM, NaCl: 300 mM, pH 7.5). After incubation at 30 °C for predetermined periods, the reaction was quenched by adding an equal volume of 1 M HCl aq., which was then analyzed by reversed-phase HPLC at a flow rate of 1.0 mL min^−1^ monitoring at 229 nm with a linear gradient of elutions (solvent A: 0.05% trifluoroacetic acid in water; solvent B: 0.05% trifluoroacetic acid in acetonitrile; percentages of solvent A: 95% at 0 min, 80% at 15 min, 30% at 115 min).

### Determination of acid dissociation constant p*K*_a_

Citrate buffers (pH 3.0, 4.0), phosphate buffers (pH 5.0, 5.5, 6.0, 6.5, 7.0, 7.5, 8.0), and boric-acid buffers (pH 8.5, 9.0, 9.5, 10.0, 10.5, 11.0, 11.5, 12.0, 12.5) were prepared for the measurements. To a quartz cell were added a thiol compound (5.0 mM, 12.5 μL) and a buffer of the desired pH (487.5 μL) at 25 °C. After mixing the sample, the pH value and absorbance at 240 nm of the resultant solution were measured. The plot of the absorbance values at each pH was analyzed by KaleidaGraph (version 5.0.1) for curve fitting to evaluate p*K*_a_.

### Determination of redox potential *E*^0^′

The *E*^0^′ value of a thiol compound was determined by following the previously reported protocol.^43^ A buffer (100 mM Tris–HCl, 1.0 mM EDTA, pH 7.0) was degassed by N_2_ bubbling for more than 1 h prior to use. DTT^red^ (60 μM, 2.5 mL) in the buffer was added to a disulfide (60 μM, 2.5 mL) in the buffer under N_2_, which was stirred at 25 ± 0.1 °C for 24 h. To quench the reaction, an aliquot of the reaction mixture (1 mL) was added to 1 M HCl aq. (200 μL), and the obtained sample solution was immediately analyzed by reversed-phase HPLC (Tosoh TSKgel ODS-100V column, *φ*4.6 × 250 mm). The column was equilibrated with water containing 0.1% trifluoroacetic acid at a flow rate of 1.2 mL min^−1^ prior to use. The RP-HPLC analysis was conducted with water containing 0.1% trifluoroacetic acid (eluent A) and CH_3_CN containing 0.1% trifluoroacetic acid (eluent B) with a linear gradient (percentage of eluent B: 0–4% in 0–10 min, 4–7% in 10–20 min). The concentrations of the species at equilibrium were calculated from the observed peak areas and corresponding calibration curves.

The equilibrium constant *K*_eq_ of the reaction (eqn (1)), described as eqn (2), was determined by averaging seven individual experiments following the above procedure.

1 Disulfide + DTT^red^ ⇌ 2Thiol + DTT^ox^

2
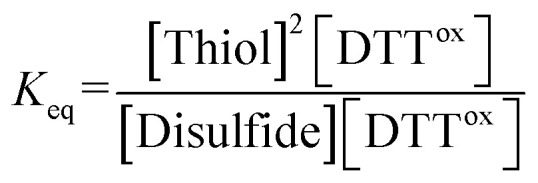


The redox potential *E*^0^′ was calculated by the Nernst equation (eqn (3)).3
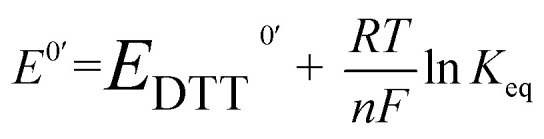
where *n* is the number of transferred electrons (*n* = 2), *F* is Faraday's constant (96 500 C mol^−1^), *R* is the universal gas constant (8.314 J K^−1^ mol^−1^), *T* is the temperature (298 K), and *E*^0^′_DTT_ is the redox potential of DTT (–327 mV).

## Conclusions

Protein misfolding is believed to be the primary cause of many common degenerative diseases. Therefore, trapping folding intermediates and misfolded forms is fundamentally important for the characterization of the disease-related biological properties of the misfolded proteins. Investigation of the folding intermediates is also important to understand the detailed mechanisms and pathways of protein folding. Currently, the methods to trap folding intermediates and misfolded forms are limited to essentially irreversible processes. In this study, we demonstrated rapid disulfide-bonding and stable formation of non-native isomers of RNase A and BPTI using cysteine-containing dipeptides, Cys-Dap and Cys-Tamp, in the presence of GSSG. Owing to the intrinsic reversibility of the disulfide bonds upon redox stimuli, the disulfide bond-based approach has great potential to develop novel reversible methodologies enabling the trapping of transient and misfolded forms of proteins by forming intermolecular disulfide-bonds and restarting the oxidative folding of the trapped forms by subsequent cleavage of the intermolecular disulfide bonds. The thiol–disulfide bond chemistry also relates to virus infection and immune suppression activities. ORF8 is an accessory protein of severe acute respiratory syndrome coronavirus 2 (SARS-CoV-2) that is proposed to interfere with immune responses.^44,45^ In the large-scale dimeric structure, ORF8 contains three sets of intramolecular disulfide bonds per monomer and a single intermolecular disulfide bond connecting the monomers. Disulfide-bond cleaving and disturbing compounds such as Cys-Dap and Cys-Tamp would be useful to investigate the mechanisms of the unique molecular structure of ORF8 to fulfill the functions, which can also lead to a drug design. Thus, the molecular design reported in this study is an important step to develop methodologies for such reversible protein folding manipulations as well as for analysis of virus infection activities.

## Conflicts of interest

There are no conflicts to declare.

## Supplementary Material

RA-012-D2RA04044A-s001

## References

[cit1] Dobson C. M. (2001). Philos. Trans. R. Soc. London, Ser. B.

[cit2] Kim Y. E., Hipp M. S., Hayer-Hartl A. B. M., Hartl F. U. (2013). Annu. Rev. Biochem..

[cit3] Anfinsen C. B. (1973). Science.

[cit4] Creighton T. E. (1997). Biol. Chem..

[cit5] Narayan M., Welker E., Wedemeyer W. J., Scheraga H. A. (2000). Acc. Chem. Res..

[cit6] Dobson C. M. (2003). Nature.

[cit7] Baneyx F., Mujacic M. (2004). Nat. Biotechnol..

[cit8] Arolas J. L., Aviles F. X., Chang J.-Y., Ventura S. (2006). Trends Biochem. Sci..

[cit9] Arai K., Iwaoka M. (2021). Molecules.

[cit10] Bucciantini M., Giannoni E., Chiti F., Baroni F., Formigli L., Zurdo J., Taddei N., Ramponi G., Dobson C. M., Stefani M. (2002). Nature.

[cit11] Haass C., Selkoe D. J. (2007). Nat. Rev. Mol. Cell Biol..

[cit12] Eisenberg D., Jucker M. (2012). Cell.

[cit13] Ninagawa S., Tada S., Okumura M., Inoguchi K., Kinoshita M., Kanemura S., Imami K., Umezawa H., Ishikawa T., B Mackin R., Torii S., Ishihama Y., Inaba K., Anazawa T., Nagamine T., Mori K. (2020). eLife.

[cit14] Creighton T. E., Darby N. J., Kemmink J. (1996). FASEB J..

[cit15] Konishi Y., Ooi T., Scheraga H. A. (1982). Proc. Natl. Acad. Sci. U.S.A..

[cit16] Goldenberg D. P. (1992). Trends Biochem. Sci..

[cit17] Hanpanich O., Maruyama A. (2020). Biomaterials.

[cit18] Nishimura T., Akiyoshi K. (2020). Bioconjugate Chem..

[cit19] Wilkinson B., Gilbert H. F. (2004). Biochim. Biophys. Acta, Proteins Proteomics.

[cit20] Lees W. J. (2008). Curr. Opin. Chem. Biol..

[cit21] Okumura M., Shimamoto S., Hidaka Y. (2012). FEBS J..

[cit22] Lukesh III J. C., Palte M. J., Raines R. T. (2012). J. Am. Chem. Soc..

[cit23] Reddy P. S., Metanis N. (2016). Chem. Commun..

[cit24] Okada S., Matsusaki M., Arai K., Hidaka Y., Inaba K., Okumura M., Muraoka T. (2019). Chem. Commun..

[cit25] Tsukagoshi S., Mikami R., Arai K. (2020). Chem.–Asian J..

[cit26] Feng H., Zhou Z., Bai Y. (2005). Proc. Natl. Acad. Sci. U.S.A..

[cit27] Neudecker P., Robustelli P., Cavalli A., Walsh P., Lundström P., Zarrine-Afsar A., Sharpe S., Vendruscolo M., Kay L. E. (2012). Science.

[cit28] Tinzl M., Hilvert D. (2021). ChemBioChem.

[cit29] Zander T., Phadke N. D., Bardwell J. C. A. (1998). Methods Enzymol..

[cit30] Scheraga H. A., Wedemeyer W. J., Welker E. (2001). Methods Enzymol..

[cit31] Miyake R., Tashiro S., Shiro M., Tanaka K., Shionoya M. (2008). J. Am. Chem. Soc..

[cit32] Miyake R., Ando A., Ueno M., Muraoka T. (2019). J. Am. Chem. Soc..

[cit33] Bierzynski A., Kim P. S., Baldwin R. L. (1982). Proc. Natl. Acad. Sci. U.S.A..

[cit34] Rothwarf D. M., Scheraga H. A. (1993). Biochemistry.

[cit35] Iwaoka M., Juminaga D., Scheraga H. A. (1998). Biochemistry.

[cit36] Weissman J. S., Kim P. S. (1991). Science.

[cit37] Kojima R., Okumura M., Masui S., Kanemura S., Inoue M., Saiki M., Yamaguchi H., Hikima T., Suzuki M., Akiyama S., Inaba K. (2014). Structure.

[cit38] Okumura M., Kanemura S., Matsusaki M., Kinoshita M., Saio T., Ito D., Hirayama C., Kumeta H., Watabe M., Amagai Y., Lee Y.-H., Akiyama S., Inaba K. (2021). Structure.

[cit39] Jocelyn P. C. (1967). Eur. J. Biochem..

[cit40] Ellman G. L. (1959). Arch. Biochem. Biophys..

[cit41] Lyles M. M., Gilbert H. F. (1991). Biochemistry.

[cit42] Laemmli U. K. (1970). Nature.

[cit43] Arai K., Ueno H., Asano Y., Chakrabarty G., Shimodaira S., Mugesh G., Iwaoka M. (2018). ChemBioChem.

[cit44] Flower T. G., Buffalo C. Z., Hooy R. M., Hurley J. H. (2021). Proc. Natl. Acad. Sci. U.S.A..

[cit45] Young B. E., Fong S.-W., Chan Y.-H., Mak T.-M., Ang L. W., Anderson D. E., Lee C. Y.-P., Amrun S. N., Lee B., Goh Y. S., Su Y. C. F., Wei W. E., Kalimuddin S., Chai L. Y. A., Pada S., Tan S. Y., Sun L., Parthasarathy P., Chen Y. Y. C., Barkham T., Lin R. T. P., Maurer-Stroh S., Leo Y.-S., Wang L.-F., Renia L., Lee V. J., Smith G. J. D., Lye D. C., Ng L. F. P. (2020). Lancet.

