# Supplementary material for: Cysteine-based protein folding modulators for trapping intermediates and misfolded forms
Source: RSC Adv. 2022 Sep 21;12(41):26658–64. doi: 10.1039/d2ra04044a (PMC9490518; doi:10.1039/d2ra04044a)
Supplement: RA-012-D2RA04044A-s001 [file RA-012-D2RA04044A-s001.pdf]

Electronic Supplementary Material (ESI) for RSC Advances.
This journal is © The Royal Society of Chemistry 2022

**Supplementary Information**

# Cysteine-based protein folding modulators for trapping intermediates and misfolded forms

Hayato Nishino,[a†] Mai Kitamura,[a†] Shunsuke Okada,[a] Ryosuke Miyake,[b] Masaki Okumura[c] and Takahiro Muraoka*[a,d,e]

[a.] *Department of Applied Chemistry, Graduate School of Engineering, Tokyo University of Agriculture and Technology, 2-24-16 Naka-cho, Koganei, Tokyo 184-8588, Japan. E-mail: muraoka@go.tuat.ac.jp*

[b.] *Department of Chemistry and Biochemistry, Graduate School of Humanities and Sciences, Ochanomizu University, 2-1-1 Otsuka, Bunkyo-ku, Tokyo 112-8610, Japan.*

[c.] *Frontier Research Institute for Interdisciplinary Sciences, Tohoku University, 6-3 Aramaki-Aza-Aoba, Aoba-ku, Sendai 980-8578, Japan.*

[d.] *Institute of Global Innovation Research, Tokyo University of Agriculture and Technology, 3-8-1 Harumi-cho, Fuchu, Tokyo 183-8538, Japan.*

[e.] *Kanagawa Institute of Industrial Science and Technology, 3-2-1 Sakato, Takatsu-ku, Kawasaki, Kanagawa 213-0012, Japan.*

[†] *These authors contributed equally to this work.*

## Table of Contents

1. **Materials**....................S2
2. **Instrumentation**....................S3
3. **Synthesis**....................S4
4. **SDS-PAGE**....................S7
5. **Reverse-phase HPLC analyses**....................S8
6. **CD spectroscopic analyses**....................S9

## 1. Materials

Deuterated solvents were purchased from Kanto Chemicals (Tokyo, Japan). Acetonitrile, 35% hydrochloric acid, and trifluoroacetic acid (TFA) were purchased from Kishida Chemical (Tokyo, Japan). Coomassie brilliant blue, *N*,*N*-diisopropylethylamine, 5,5'-dithiobis(2-nitrobenzoic acid) (DTNB), L-glutathione oxidized (GSSG), L-glutathione reduced (GSH), and guanidine hydrochloride (GdnHCl) were purchased from Nacalai Tesque (Kyoto, Japan). α-Cyano-4-hydroxycinnamic acid, cytidine 2':3'-cyclic monophosphate monosodium salt (cCMP), malPEG-2000, ribonuclease A (RNaseA) from bovine pancreas, and triethylamine were purchased from Sigma-Aldrich (St. Louis, MO, USA). Bovine pancreatic trypsin inhibitor (BPTI) was purchased from ProSpec (Rehovot, Israel). (*S*)-3-Amino-2-(*tert*-butoxycarbonylamino)propanoic acid, *N*-(*tert*-butoxycarbonyl)-*S*-trityl-L-cysteine, 4-(4,6-dimethoxy-1,3,5-triazin-2-yl)-4-methylmorpholinium chloride, 1,1,1,3,3,3-hexafluoro-2-propanol, and triisopropylsilane were purchased from Tokyo Chemical Industry (Tokyo, Japan). Column chromatography was carried out with Silica Gel 60 (spherical, neutral, particle size: 63–210 μm) purchased from Kanto Chemicals. Deionized water (filtered through a 0.22 μm membrane filter, >18.2 MΩ cm) was purified in Purelab DV35 of ELGA (Buckinghamshire, UK) and a Milli-Q system of Merck Millipore (Burlington, MA, USA).

## 2. Instrumentation

Nuclear magnetic resonance (NMR) spectra were recorded on a JNM-ECA 500 spectrometer (500 MHz for $^1$H) of JEOL (Tokyo, Japan), where the chemical shifts ($\delta$) were determined with respect to the resonances corresponding to the residual non-deuterated solvent for $^1$H ($CDCl_3$: 7.24 ppm, $D_2O$: 4.67 ppm). Matrix-assisted laser desorption/ionization time-of-flight (MALDI-TOF MS) was performed on autoflex speed spectrometer of Bruker (Bremen, Germany). High-resolution electrospray ionization (HR ESI) TOF MS spectra were recorded on micrOTOF-Q II-S1 of Bruker (Bremen, Germany). Analytical thin layer chromatography (TLC) was performed on precoated, glass-backed silica gel Merck 60 F254. Visualization of the developed chromatogram was performed by UV absorbance, Hanessian's stain or iodine. UV-vis absorption spectra were recorded on V-750 UV-Vis spectrophotometer of JASCO (Tokyo, Japan). Analytical reversed-phase high-performance liquid chromatography (RP-HPLC) was conducted with Primaide HPLC system of HITACHI (Tokyo, Japan) using TSKgel Protein C4-300 column of Tosoh Bioscience ($\varphi$4.6 × 150 mm, Tokyo, Japan). Semi-preparative reversed-phase high-performance liquid chromatography performed on PU-4086-Binary pump, UV-4075 detector and CHF122SC fraction collector of JASCO (Tokyo, Japan) attached with TA12S05-2520WX Actus Triart column of YMC ($\varphi$20 × 250 mm, Tokyo, Japan).

## 3. Synthesis

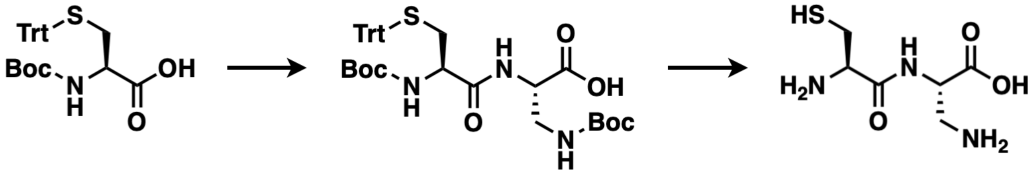


**Scheme 1.** Synthetic scheme of Cys-Dap.

**Synthesis of Cys-Dap.** To an anhydrous MeOH (5 mL) solution of *N*-(*tert*-butoxycarbonyl)-*S*-trityl-L-cysteine (**1**, 681 mg, 1.47 mmol) were added 4-(4,6-dimethoxy-1,3,5-triazin-2-yl)-4-methylmorpholinium chloride (406 mg, 1.47 mmol), *N*,*N*-diisopropylethylamine (252 μL, 1.47 mmol) and (*S*)-3-amino-2-(*tert*-butoxycarbonylamino)propanoic acid (200 mg, 0.979 mmol) under $N_2$ at 0 °C. After stirring for 15 min at 0 °C followed by 20 h at 25 °C, the reaction mixture was evaporated to dryness. $CHCl_3$ (50 mL) was added to the residue, and the organic mixture was washed with 0.5 M HCl aq. (50 mL), saturated $NaHCO_3$ aq. (50 mL), water (50 mL) and brine (50 mL). The organic layer was dried over $Na_2SO_4$, and the mixture was filtered off from insoluble substances. The filtrate was evaporated to dryness, and the residue was chromatographed with silica gel with an eluent of $CHCl_3$, MeOH, and AcOH mixture (90/10/0.1) to allow for isolation of **2** (269 mg, 0.414 mmol) in 42% yield.

To a 1,1,1,3,3,3-hexafluoro-2-propanol (2.296 mL) solution of **2** (129 mg, 0.414 mmol) and triisopropylsilane (24 μL, 0.058 mmol) was added concentrated HCl aq. (1.68 mL, 19.0 mmol) at 0 °C. After stirring for 15 min at 0 °C followed by 20 h at 25 °C, to the reaction mixture was added water (30 mL). The aqueous mixture was washed with $CHCl_3$ (20 mL, three times). The aqueous layer was evaporated to dryness, and the residue was chromatographed with a reverse-phase high performance liquid chromatography (HPLC) with an eluent of water, acetonitrile, and trifluoroacetic acid mixture (100/0/0.1 to 80/20/0.1) to allow isolation of Cys-Dap (12 mg, 0.058 mmol) in 14% yield.

Characterization of **2**. TLC ($CHCl_3$/MeOH/AcOH = 90/10/0.1): $R_f$ = 0.23 visualized with ninhydrin coloration; $^1$H NMR (500 MHz, $CDCl_3$, 25 °C): $\delta$ = 7.40–7.38 (m, 6H), 7.27–7.24 (m, 6H), 7.20–7.17 (m, 4H), 5.78 (brs, 1H), 5.29 (brs, 1H), 4.17 (brs, 1H), 3.84 (brs, 1H), 3.52 (brs, 2H), 2.56 (brs, 2H), 1.38 (s, 18H) ppm; HR ESI-TOF MS: *m/z* = 672.2723 (calculated *m/z* on the basis of the monoisotopic mass of $C_{35}H_{43}N_3NaO_7S$ $[M + Na]^+$ = 672.2719).

Characterization of Cys-Dap. $^1$H NMR (500 MHz, $D_2O$, 25 °C): $\delta$ = 4.23 (t, $J$ = 6.0 Hz, 1H), 4.15 (t, $J$ = 5.5 Hz, 1H), 3.83 (d, $J$ = 6.0 Hz, 2H), 3.12–3.01 (m, 2H) ppm; MALDI-TOF MS: $m/z$ = 208.011 (calculated $m/z$ on the basis of the monoisotopic mass of $C_6H_{14}N_3O_3S$ $[M + H]^+$ = 208.075).

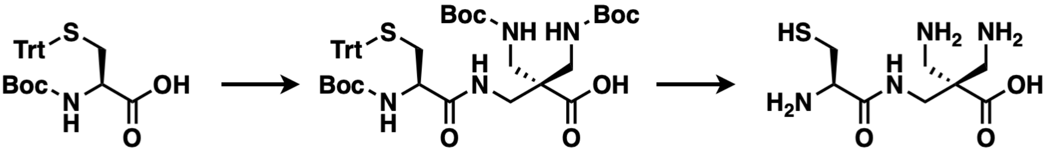


**Scheme 2.** Synthetic scheme of Cys-Tamp.

**Synthesis of Cys-Tamp.** To an anhydrous EtOH (3 mL) solution of *N*-(*tert*-butoxycarbonyl)-*S*-trityl-L-cysteine (**1**, 276 mg, 0.595 mmol) were added 4-(4,6-dimethoxy-1,3,5-triazin-2-yl)-4-methylmorpholinium chloride (165 mg, 0.596 mmol), triethylamine (82.3 µL, 0.594 mmol) and 3-amino-2,2-bis(*tert*-butoxycarbonylaminomethyl)propanoic acid (138 mg, 0.396 mmol) under $N_2$ at 0 °C. After stirring for 15 min at 0 °C followed by 20 h at 25 °C, the reaction mixture was evaporated to dryness. $CHCl_3$ (50 mL) was added to the residue, and the organic mixture was washed with 0.5 M HCl aq. (50 mL), saturated $NaHCO_3$ aq. (50 mL), water (50 mL) and brine (50 mL). The organic layer was dried over $Na_2SO_4$, and the mixture was filtered off from insoluble substances. The filtrate was evaporated to dryness, and the residue was chromatographed with silica gel with an eluent of $CHCl_3$, MeOH, and AcOH mixture (90/10/0.1) to allow for isolation of **3** (169 mg, 0.213 mmol) in 54% yield.

To a 1,1,1,3,3,3-hexafluoro-2-propanol (1.148 mL) solution of **3** (169 mg, 0.213 mmol) and triisopropylsilane (12 µL, 0.029 mmol) was added concentrated HCl aq. (840 µL, 9.5 mmol) at 0 °C. After stirring for 15 min at 0 °C followed by 20 h at 25 °C, to the reaction mixture was added water (30 mL). The aqueous mixture was washed with $CHCl_3$ (20 mL, three times). The aqueous layer was evaporated to dryness, and the residue was chromatographed with a reverse-phase HPLC with an eluent of water, acetonitrile, and trifluoroacetic acid mixture (100/0/0.1 to 80/20/0.1) to allow isolation of Cys-Tamp (43 mg, 0.17 mmol) in 80% yield.

Characterization of **3**. TLC ($CHCl_3$/MeOH/AcOH = 90/10/0.1): $R_f$ = 0.43 visualized with ninhydrin coloration; $^1$H NMR (500 MHz, $CDCl_3$, 25 °C): $\delta$ = 7.43–7.39 (m, 6H), 7.30–7.27

(m, 6H), 7.22 (brs, 2H), 4.14 (brs, 1H), 4.03 (s, 1H), 3.70 (s, 1H), 3.23 (brs, 3H), 2.54 (brs, 3H), 1.41 (s, 27H) ppm; MALDI-TOF MS: *m/z* = 815.390 (calculated *m/z* on the basis of the monoisotopic mass of $C_{42}H_{56}N_4NaO_9S$ $[M + Na]^+$ = 815.366).

Characterization of Cys-Tamp. $^1H$ NMR (500 MHz, $D_2O$, 25 °C): $\delta$ = 4.28 (t, $J$ = 5.5 Hz, 1H), 3.84–3.67 (m, 4H), 3.36–3.26 (m, 4H), 2.91 (t, $J$ = 6.5 Hz, 1H) ppm; MALDI-TOF MS: *m/z* = 251.122 (calculated *m/z* on the basis of the monoisotopic mass of $C_8H_{19}N_4O_3S$ $[M + H]^+$ = 251.117).

## 4. SDS-PAGE

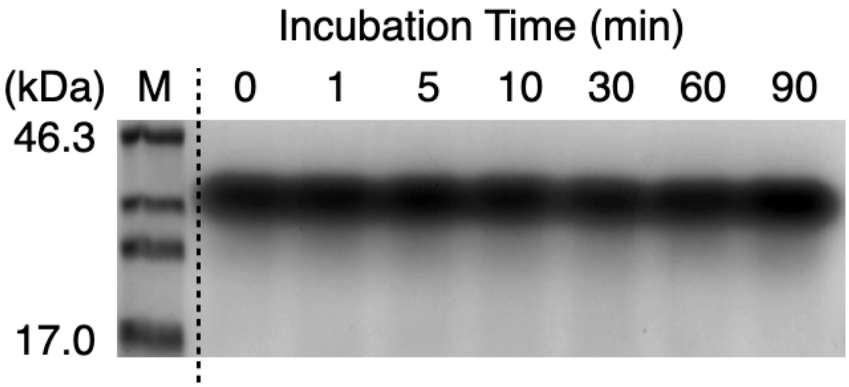


**Figure S1** An SDS-PAGE gel image of Cys-Tamp (1.0 mM) dissolved in a buffer (50 mM Tris-HCl, 300 mM NaCl, pH 7.5) containing malPEG-2000.

## 5. Reverse-phase HPLC analyses

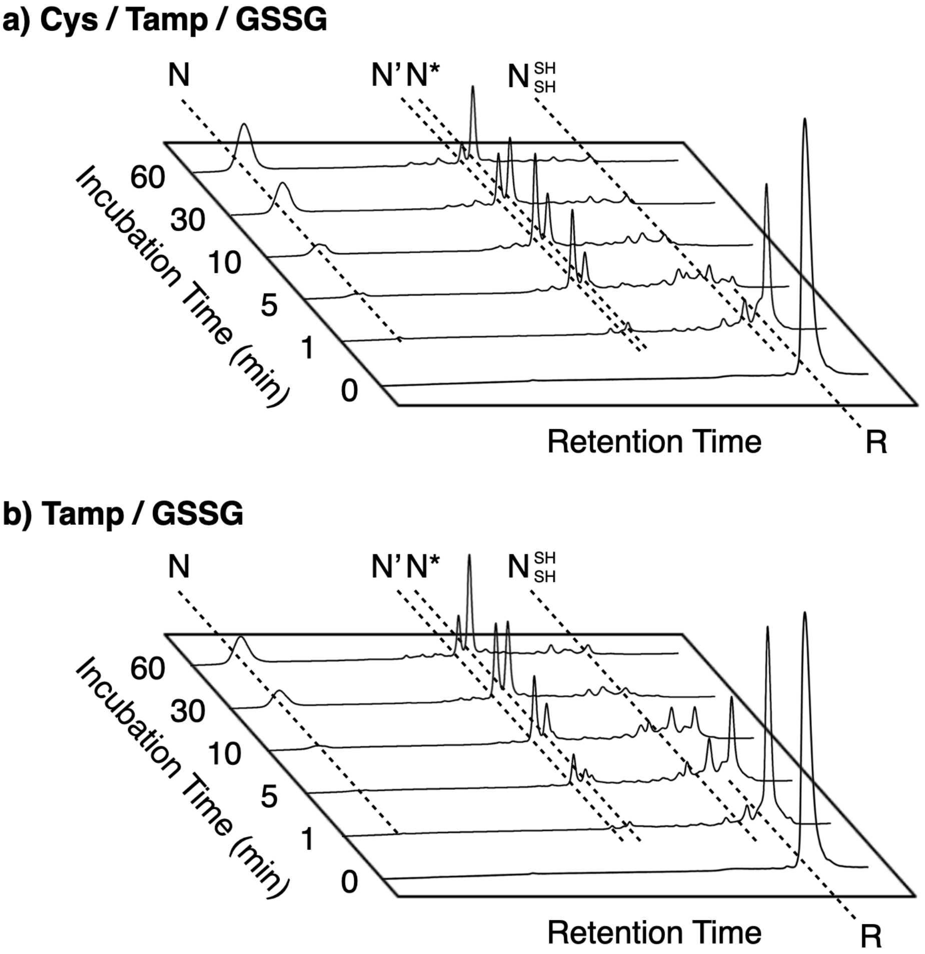


**Figure S2** Time-course reverse-phase HPLC traces of oxidative folding of BPTI (30 μM) in the presence of (a) cysteine, Tamp and GSSG, and (b) Tamp and GSSG (cysteine, Tamp: 1.0 mM, GSSG: 0.20 mM) between 16- and 45-min retention time. N and R depict native and reduced forms of BPTI, respectively. The yields of N at 60 min were a) 41% and b) 23%. Eluent buffers: water (containing 0.05% TFA) and $CH_3CN$ (containing 0.05% TFA) with a linear gradient (water/$CH_3CN$ = 80/20 at 16 min to 65/35 at 45 min); flow rate: 1.0 mL $min^{-1}$; detection wavelength: 229 nm; temperature: 25 °C.

## 6. CD spectroscopic analyses

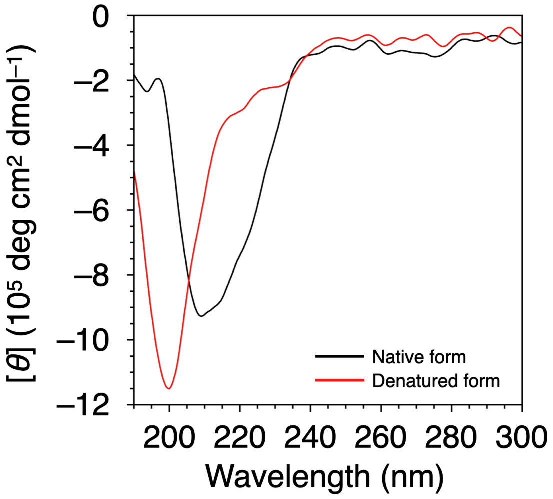


**Figure S3** Circular dichroism (CD) spectra of RNase A (8.0 μM) in the native form (black line, 50 mM Tris-HCl, 300 mM NaCl, pH 7.5) and in the denatured form after the dialysis process (red line, 10 mM HCl) at 25 °C. Optical length: 1.0 mm.
